# Orolabial Herpes Simplex Virus Type 2 Infection: A Case Report Highlighting the Importance of Viral Typing

**DOI:** 10.1002/ccr3.73334

**Published:** 2026-08-17

**Authors:** Yujie Chu, Caixing Liu, Yinfeng Cui, Yan Biao, Lei Wang, Shengyin Hong

**Affiliations:** ^1^ Department of Stomatology The Affiliated Hospital of Yanbian University (Yanbian Hospital) Yanji Jilin China; ^2^ Department of Stomatology College of Medicine, Yanbian University Yanji Jilin China

**Keywords:** atypical presentation, case report, herpes simplex virus type 2, orolabial herpes, type‐specific serology

## Abstract

HSV‐2 can present as atypical orolabial lesions and may be misdiagnosed as HSV‐1. Viral typing is essential in atypical cases to ensure accurate diagnosis, appropriate management, and effective patient counseling.

## 1 Introduction

Herpes simplex virus (HSV) infection is highly prevalent worldwide, affecting a large proportion of the global population [1, 2, 3]. HSV‐1 is traditionally associated with orolabial disease, whereas HSV‐2 is primarily linked to genital infection [4]. However, changing sexual practices have increasingly blurred this distinction [5].

Orolabial HSV‐2 infection is uncommon and may be overlooked in clinical practice due to site‐based diagnostic assumptions. This case highlights the importance of considering HSV‐2 in atypical orolabial lesions and the role of viral typing in accurate diagnosis and management.

## 2 Case Presentation

A 24‐year‐old woman presented with a one‐week history of recurrent vesicles, ulceration, crusting, and associated pruritus on both her upper and lower lip. The patient had no history of diabetes mellitus, immunodeficiency, chronic systemic disease, or immunosuppressive medication use. No genital lesions or other mucocutaneous abnormalities were identified during physical examination. After laboratory evaluation demonstrated type‐specific HSV‐2 seropositivity with negative HSV‐1 serology, a presumptive diagnosis of HSV‐2‐associated orolabial infection was established. Oral valacyclovir (500 mg twice daily for 14 days) was then initiated, together with topical penciclovir and supportive local therapy.

### 2.1 History

One week prior to presentation, the patient developed lesions on the vermilion border of both lips without an identifiable precipitating factor. The lesions initially presented as multiple, scattered, pinpoint to small vesicles, accompanied by significant pruritus. These vesicles ruptured spontaneously within a short period, releasing clear fluid and subsequently forming white or yellowish crusts. Notably, new vesicles repeatedly emerged around partially healed lesions, resulting in a protracted and relapsing course.

The patient attempted self‐treatment with topical valacyclovir ointment, with no significant clinical improvement. Since symptom onset, she reported no systemic manifestations such as fever or malaise and denied severe local pain.

### 2.2 Past Medical History

The patient was previously healthy and denied a history of recurrent herpes labialis. There was no recent history of systemic medication use or trauma, and she denied known allergies to cosmetics or food.

### 2.3 Physical and Specialized Examination

Examination of the affected area (Figure 1) revealed multiple scattered vesicles measuring approximately 1–2 mm in diameter on the vermilion mucosa and border of both lips. Some vesicles contained clear fluid, while others had ruptured, forming superficial erosions covered by thin white or yellowish crusts. Mild erythema was noted in the surrounding skin. No palpable enlargement of the submandibular or cervical lymph nodes was observed.

**FIGURE 1:**
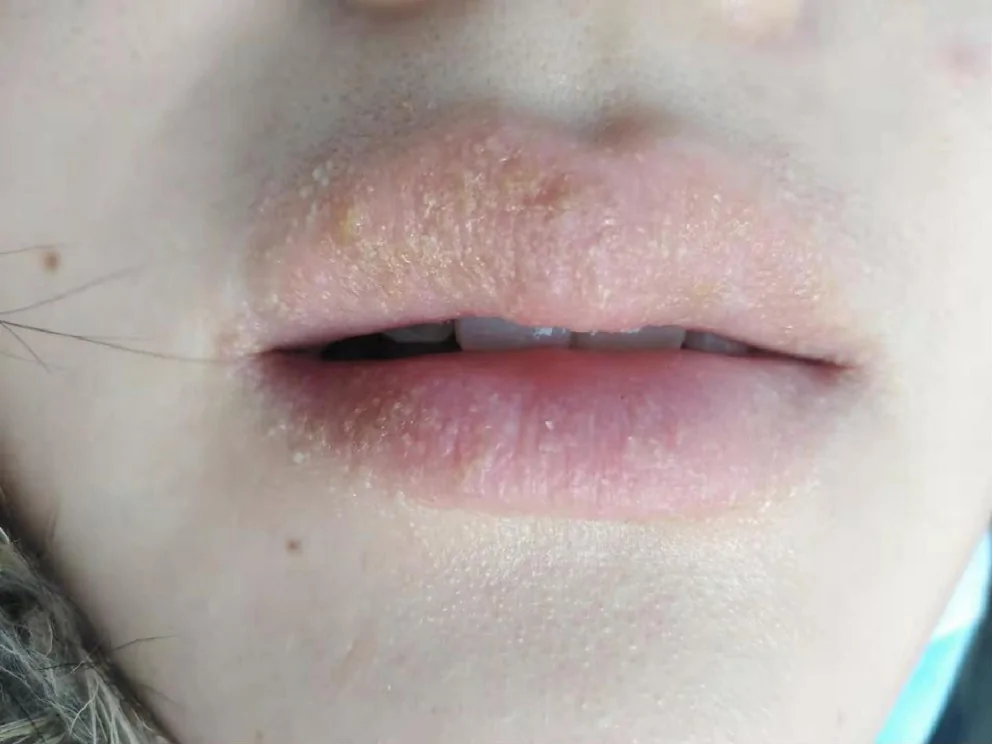
Initial presentation of orolabial lesions. Multiple small vesicles and superficial erosions involving the upper and lower vermilion border at first presentation.

## 3 Investigations

Because recurrent herpes labialis is most commonly caused by HSV‐1, the initial clinical impression was HSV‐1 infection based on the lesion location. However, the lack of improvement following self‐administered topical antiviral therapy prompted further diagnostic evaluation. At this stage, alternative diagnoses, including HSV‐2 infection, aphthous ulceration, contact cheilitis, and bacterial infection, were considered. Given the atypical clinical course and the possibility that lesion location alone could not reliably distinguish HSV type, type‐specific HSV‐1 and HSV‐2 serological testing was performed to further clarify the diagnosis. Type‐specific serological testing was performed to clarify the etiology (Table 1). The results were as follows: a markedly elevated herpes simplex virus type 2 IgM level (HSV‐2 IgM: 43.38 AU/mL; reference range: 0–6 AU/mL) and a positive HSV‐2 IgG level (186.51 AU/mL; reference range: 0–14 AU/mL). No significant elevation in herpes simplex virus type 1–specific antibodies was detected.

**TABLE 1:** Serological findings in the patient with orolabial herpes lesions.

Test	Result	Unit	Reference range	Interpretation
HSV‐2 IgM	43.38	AU/mL	0–6	Positive
HSV‐2 IgG	186.51	AU/mL	0–14	Positive
HSV‐1 IgM	Negative	AU/mL	0–6	Negative
HSV‐1 IgG	Negative	AU/mL	0–14	Negative
Cytomegalovirus IgG	2135.93	AU/mL	0–10	Past infection
Rubella virus IgG	22.46	IU/mL	0–5	Past immunity

Abbreviations: HSV, herpes simplex virus; IgG, immunoglobulin G; IgM, immunoglobulin M.

In addition, cytomegalovirus IgG was positive (2135.93 AU/mL), consistent with past infection, and rubella virus IgG was positive (22.46 IU/mL), indicating prior immunity or exposure. All other serological parameters were within normal limits.

Taken together with the clinical presentation, HSV‐2 IgG positivity indicates prior or latent infection, whereas markedly elevated HSV‐2 IgM suggests recent viral reactivation and active replication. Taken together with the clinical presentation, these findings were considered consistent with presumptive HSV‐2‐associated orolabial infection.

Because HSV PCR testing was not routinely available at our institution, we only established presumptive HSV‐2 infection. The diagnosis was established based on the characteristic clinical manifestations, positive HSV‐2 type‐specific serological findings, negative HSV‐1 serology, and complete clinical response to systemic antiviral therapy. Differential diagnoses included recurrent HSV‐1 infection, impetigo, allergic contact cheilitis, aphthous disease, and erythema multiforme. These conditions were considered less likely because of the lesion morphology, laboratory findings, clinical course, and treatment response.

## 4 Differential Diagnosis

The differential diagnosis for recurrent vesicular and erosive lesions of the lips includes herpes simplex virus type 1 (HSV‐1) infection, allergic contact cheilitis, impetigo, and erosive lichen planus.

HSV‐1 infection was initially considered given the typical orolabial distribution; however, the absence of HSV‐1–specific antibodies and the presence of markedly elevated HSV‐2 IgM and IgG made HSV‐1 infection unlikely.

Allergic contact cheilitis was considered due to the presence of pruritus; however, the recurrent vesicular morphology, spontaneous rupture with clear fluid, and favorable response to antiviral therapy were not consistent with a purely inflammatory or allergic condition.

Impetigo was excluded based on the absence of honey‐colored crusts, systemic symptoms, and negative bacterial features.

Erosive lichen planus was considered but deemed unlikely given the acute onset, vesicular presentation, and lack of characteristic reticular white striae.

Overall, the clinical features combined with type‐specific serological findings supported a diagnosis of active orolabial HSV‐2 infection.

## 5 Treatment

Following establishment of presumptive HSV‐2 infection, the patient was treated with combined systemic and topical antiviral therapy. Oral valacyclovir was prescribed at a dose of 500 mg twice daily for 14 days, and topical penciclovir cream was applied as a thin layer to the affected areas 4–5 times daily over the same period. The clinical course is summarized in Timeline (Table 2).

**TABLE 2:** Timeline illustrating the evolution of clinical manifestations, diagnostic reasoning, laboratory evaluation, and treatment course.

Time point	Clinical course and diagnostic reasoning
Day 1 of onset	Vesicles and erosions developed on the upper and lower vermilion border
Day 4 of onset	Self‐administered topical antiviral therapy without clinical improvement
Day 7 of onset	Presented to hospital. Based on the lesion location, recurrent HSV‐1 infection was initially considered
Day 7 of onset	Because of the poor response to empirical therapy and the atypical clinical course, the differential diagnosis was reconsidered. Type‐specific HSV‐1 and HSV‐2 serological testing was requested
Day 8 of onset (Day 0 of treatment)	Serological findings were consistent with HSV‐2 infection. A presumptive diagnosis of HSV‐2‐associated orolabial infection was established, and oral valacyclovir therapy was initiated
Day 2 of treatment	No new vesicles developed. Existing vesicles became flattened with reduced exudation
Day 3 of treatment	Extensive crust formation with marked improvement in lip erythema, swelling, and pain
Day 7 of treatment	Most crusts had detached, and the lesions had almost completely healed
Two‐week follow‐up	Complete resolution of lesions with no recurrence observed during the available follow‐up period

## 6 Outcome and Follow‐Up

At a follow‐up visit two weeks after initiation of antiviral therapy, the vesicular and erosive lesions had completely resolved, with no evidence of new blister formation (Figure 2). Mild diffuse erythema of the perioral skin was noted; however, there was no associated pain, ulceration, or crusting.

**FIGURE 2:**
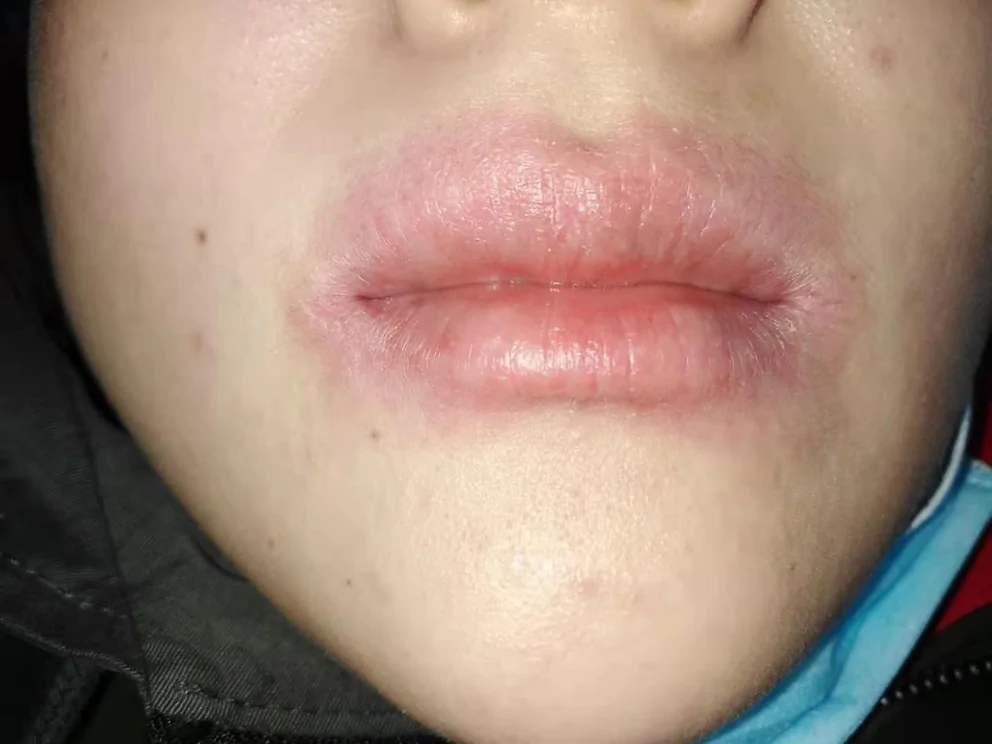
Follow‐up after treatment. Resolution of vesicular and erosive lesions of the upper and lower lips after antiviral therapy, with mild residual erythema.

Supportive treatment was subsequently initiated to address residual inflammation. Wet compresses with sodium bicarbonate solution were applied for one week, and topical fluocinolone acetonide ointment was administered once to twice daily for one week. The perioral erythema gradually subsided, and the patient reported further symptomatic improvement.

No additional follow‐up visits were conducted. According to available clinical records and patient self‐report, no recurrence was observed during the available two‐week follow‐up period.

## 7 Patient's Perspective

The patient reported that the recurrent lip lesions caused significant discomfort and anxiety, particularly due to the lack of improvement with initial treatment. She felt relieved after receiving a clear diagnosis and understanding the cause of her condition. Following antiviral therapy, she noted rapid improvement of symptoms and expressed satisfaction with the treatment outcome.

## 8 Discussion

Initially, the lesion location suggested HSV‐1 because HSV‐1 is the predominant cause of recurrent orolabial herpes. However, the lack of improvement after initial empirical treatment, together with negative HSV‐1 serology and positive HSV‐2 type‐specific serology, prompted reconsideration of the diagnosis. This diagnostic reassessment led clinicians to broaden the differential diagnosis and perform type‐specific HSV testing, ultimately supporting a presumptive diagnosis of HSV‐2‐associated orolabial infection. This case illustrates the importance of reassessing the initial diagnostic impression when clinical evolution and laboratory findings are discordant.

This case demonstrates that HSV‐2 can present as orolabial disease and may be misclassified as HSV‐1 if viral typing is not performed [6, 7]. PCR remains the diagnostic gold standard for HSV infection [8]. However, its sensitivity depends on lesion stage, and it may not always be feasible. In settings where PCR is unavailable, type‐specific serology interpreted together with compatible clinical findings may provide useful diagnostic support [9, 10, 11]. Accurate viral typing has important clinical implications. HSV‐2 is associated with higher recurrence rates and asymptomatic viral shedding in genital infection, whereas recurrent orolabial herpes is more commonly caused by HSV‐1 [10, 11, 12, 13]. Misclassification may therefore result in inadequate patient counseling. Systemic antiviral therapy remains effective for HSV infection regardless of anatomical site [14, 15, 16]. Clinicians should avoid relying solely on anatomical location when diagnosing HSV infection and should consider HSV‐2 in atypical orolabial presentations [17]. When empirical treatment fails or when clinical findings are inconsistent with the initial diagnostic impression, clinicians should broaden the differential diagnosis and consider type‐specific viral testing where available to avoid diagnostic delay. HSV infections can present with a broad spectrum of clinical manifestations beyond typical mucocutaneous lesions [17, 18]. To our knowledge, reports of isolated orolabial presumptive HSV‐2 infection identified by type‐specific serology without concurrent genital involvement remain limited, highlighting the clinical relevance of this case.

Although PCR remains the diagnostic gold standard for HSV infection, it was not routinely available in our institution. In this case, the diagnosis was supported by the combination of characteristic clinical manifestations, type‐specific HSV‐2 serology, negative HSV‐1 serology, and complete clinical resolution following antiviral therapy. Following type‐specific serological testing, the clinical findings were considered to be consistent with presumptive HSV‐2‐associated orolabial infection. Based on this diagnostic assessment, the patient was treated with combined systemic and topical antiviral therapy. Oral valacyclovir was selected in accordance with current guideline recommendations for HSV infection. Topical penciclovir and supportive local therapy were used as adjunctive measures to relieve symptoms and promote lesion healing rather than as substitutes for systemic antiviral treatment. The patient's complete recovery at follow‐up further supports the effectiveness of this management strategy.

This case has several limitations. First, HSV PCR testing was unavailable, and the diagnosis relied on compatible clinical findings together with type‐specific serological results. Second, information regarding sexual history, partner evaluation and counseling, and HIV/STI screening was unavailable, which limited a more comprehensive assessment of potential risk factors and patient management. Third, because this is a single case report, the findings cannot be generalized to all patients with atypical orolabial HSV‐2 infection. Nevertheless, despite these limitations, the combination of compatible clinical manifestations, type‐specific serology, and favorable clinical response to antiviral therapy provides clinically meaningful evidence supporting the presumptive diagnosis. This case highlights the importance of avoiding assumptions regarding HSV type based solely on lesion location. Greater awareness of atypical orolabial HSV‐2 infection may facilitate earlier diagnosis, appropriate antiviral treatment, and improved patient counseling.

## 9 Conclusion

This case highlights that HSV‐2 should be considered in patients presenting with atypical or recurrent orolabial lesions, particularly when clinical findings are inconsistent with HSV‐1 infection. Careful clinical reassessment together with appropriate viral typing can improve diagnostic accuracy and guide patient counseling.

## Author Contributions

**Yinfeng Cui:** supervision, conceptualization, resources, project administration. **Lei Wang:** formal analysis, writing – review and editing. **Yujie Chu:** data curation, formal analysis, investigation, writing – original draft. **Shengyin Hong:** resources, writing – review and editing. **Caixing Liu:** conceptualization, formal analysis, writing – review and editing. **Yan Biao:** data curation, investigation.

## Funding

The authors have nothing to report.

## Consent

Written informed consent for publication of this case report and accompanying images was obtained from the patient.

## Conflicts of Interest

The authors declare no conflicts of interest.

## Data Availability

The data that support the findings of this study are available from the corresponding author upon reasonable request.
