# Correction to “Orolabial Herpes Simplex Virus Type 2 Infection: A Case Report Highlighting the Importance of Viral Typing”

**DOI:** 10.1002/ccr3.73453

**Published:** 2026-08-31

Y. Chu , C. Liu , Y. Cui , B. Yan , L. Wang , S. Hong , “Orolabial Herpes Simplex Virus Type 2 Infection: A Case Report Highlighting the Importance of Viral Typing,” Clinical Case Reports 14, 2026: e73334, 10.1002/ccr3.73334.42614601 PMC13482008

The first and last names of author Yan Biao were swapped and have been updated in the author list and the Author Contributions section.

The authors' affiliation was incorrect and has been corrected to “Department of Stomatology, The Affiliated Hospital of Yanbian University (Yanbian Hospital), Yanji, Jilin, China.”

An additional affiliation for authors Yinfeng Cui and Lei Wang was inadvertently omitted. The affiliation “Department of Stomatology, College of Medicine, Yanbian University, Yanji, Jilin, China” has been added.

The online version of this article has been corrected accordingly.

We apologize for these errors.

